# How Many Thymic Epithelial Cells Are Necessary for a Proper Maturation of Thymocytes?

**DOI:** 10.3389/fimmu.2021.618216

**Published:** 2021-03-08

**Authors:** Sara Montero-Herradón, Javier García-Ceca, Agustín G. Zapata

**Affiliations:** ^1^Department of Cell Biology, Faculty of Biology, Complutense University of Madrid, Madrid, Spain; ^2^Health Research Institute, Hospital 12 de Octubre (imas12), Madrid, Spain

**Keywords:** thymic epithelial cells, thymocyte education, Eph, ephrin, regulatory T-cells

## Introduction

The central tolerance to self-antigens is achieved in the thymus through a complex process in which developing thymocytes (T) sequentially interact with thymic epithelial cells (TECs) in a 3D network histologically organized in a cortex and a medulla (1). This T-TEC crosstalk is assumed to be essential for performing the named thymocyte education (2).

## Is T-Cell Maturation Possible in an Altered Thymic Epithelial Microenvironment?

There are examples in the literature showing that profound alterations of the thymic epithelial organization, which course with total or partial absence of T-TEC interactions mediated by distinct experimental conditions, do no result in important changes in the phenotype of T-cell subsets or in a breakage of central tolerance.

Revest et al. (3) demonstrated that FGF signaling was essential for TEC proliferation and that its lack coursed with low numbers of TECs that presumably would established reduced T-TEC interactions. However, in these conditions, phenotypical intrathymic lymphoid cell differentiation occurred normally, producing both DP and SP thymocytes. Authors remarked that even a low number of TECs would establish sufficient T-TEC interactions for supporting, at least phenotypical, lymphoid cell maturation. In a similar way, mice deficient in p63, a homolog of the p53 tumor suppressor gene involved in the morphogenesis and maintenance of thymic epithelium and epidermis, exhibit hypoplasia with increased proportions of apoptotic cells but normal T-cell development (4).

The Kremen (Krm) family of proteins that interact with the secreted Wnt regulator Dickkopf (Dkk) is considered a specific inhibitor of the canonical Wnt signaling pathway (5, 6). The loss of Krm1, that results in an excessive canonical Wnt signaling, induces profound alterations of the thymic architecture including incomplete separation of cortex and medulla, big epithelial free areas (EFAs), increased numbers of immature K5^+^K8^+^ TECs and loss of cell processes in cortical (c) TECs, that undoubtedly affect the T-TEC crosstalk. However, the lack of Krm1 gene had no effects on the numbers and proportions of thymocytes (7). Accordingly, authors speculated that the histological organization of the thymus might be less relevant for the T-cell maturation than the mere occurrence of different TEC subsets. On the other hand, the presence of small groups of TECs properly organized from a histological view and expressing key molecules involved in the functional crosstalk between TECs and thymocytes would be sufficient to allow the thymocyte differentiation in mutant thymuses (3, 7). In addition, Benz and colleagues (8) used other experimental approach to demonstrate that in CCR9-deficient thymuses DN2 and DN3 thymocytes did not migrate and occupy the subcapsular cortex but developed normally, questioning the relevance of thymic niches that could just optimize the T-cell maturation rather than being a mandatory requisite for achieving the process. More recently, Cosway et al. (9) reported maintenance of T-cell tolerance in mice with a specific deletion of lymphotoxin β receptor (LTβR) gene in TECs that exhibited profound alterations of thymic medulla. In this case, dendritic cells (DCs) rather than medullary TECs appeared to be supporting the negative selection (10).

## The Condition of Ephb-Deficient Thymuses

In the last years, we have analyzed the role of tyrosine kinase receptors of the family Eph (Erythropoietin-producing hepatocyte) and their ligands ephrins (Eph receptor-interacting proteins) in the biology of the thymus. Eph-ephrin signaling participates in numerous processes in the immune and other tissues through the modulation of cell attachment, detachment, migration, proliferation, differentiation and cell fate (11). Our results, which have been summarized several times (12–14), confirm a role for EphB2, EphB3, ephrin-B1, and ephrin-B2 in the development and homeostasis of thymic epithelium. In these studies, as in those previously reported, we demonstrated that altered thymic epithelial network, which partially blocked the T-TEC interactions, does not course irrefutably with remarkable changes in the functional maturation of thymocytes, including both positive and negative selection (13).

On the other hand, the absence of these molecules clearly affected the T-TEC interactions. The mere morphological analysis of EphB-deficient thymuses suggested difficulties for establishing T-TEC contacts: scattered at random medullary epithelial islets, existence of large EFAs, retracted and disappeared epithelial cell processes (12). On the other hand, lymphoid progenitor cells differentiate in grafted EphB-deficient thymic lobes (15) and *in vitro* treatment of thymic reaggregates (RTOCs) with anti-EphB2 or anti-EphB3 specific antibodies shortened the TEC processes (13). The named thymic nurse cells (TNC) are cell complexes consisting of a single cTEC that homes 7–50 thymocytes and constitute specialized thymic niches for T-cell maturation (16). TNCs derived from EphB-deficient thymuses exhibit significantly reduced numbers of the most frequent ones containing 6–10 thymocytes (13). In fact, Eph and ephrin modulate both kinetic of cell conjugated established between DP thymocytes and isolated TECs and the formation of functional immunological synapsis (17, 18). Together all these results justify that the lack of Eph and ephrin signals alters the cellular positioning making it impossible that, as reported in other tissues, for thymocytes and TECs to intermingle and interact.

In agreement with all these data, we recently analyzed the T-cell populations of EphB2- or EphB3-deficient mice observing no phenotypical changes in the proportions of T-cell subsets phenotypically defined by specific cell markers (13, 19). Previously, we reported delayed maturation of DN (CD4^−^CD8^−^) cells but no variations in the proportions of both DP (CD4^+^CD8^+^) and SP thymocytes (CD4^+^CD8^−^ and CD4^−^CD8^+^) (20) and an analysis on the EphB-deficient TCR repertoire only found increased percentages of Vβ3^+^CD4^+^ cells in thymus and lymph nodes (14). In periphery, no changes occurred in the proportions of mutant Th1, Th2, and Th17 as compared to the values of WT cell populations, and only regulatory T-cells (Treg) of the inguinal lymph nodes, but not of spleen or thymus, showed significantly higher values as compared to WT ones (13, 19). Remarkably, the evaluation of the frequencies of positive and negative selected thymocytes of EphB-deficient thymuses did not exhibit significant differences with respect to WT proportions (13, 19). Nevertheless, as demonstrated in other experimental models (9), a role for thymic DCs in the maintenance of T-cell tolerance in all these mutants cannot be discarded.

## Changes in the Number of TECs Affect Both Thymic Growth and Thymocyte Differentiation

In order to test the hypothesis that reduced T-TEC crosstalk would support thymocyte differentiation, as others had pointed out (21, 22), we recently analyzed by flow cytometry the T-cell maturation in RTOCs containing different numbers of E14.5 thymic stromal cells (TSCs) (1–0.085 × 10^6^) 1 month after grafting under the kidney capsule of FoxN1^−/−^ mice (19). Reaggregates were performed from E14.5 WT thymic lobes receiving for 7 days 2′-dGuo that eliminate any dividing thymic cell. At 1 month, RTOCs established with 1–0.5 × 10^6^ stromal cells grew *in vivo* around 5–7 times, but those containing lower numbers of TSC yielded significantly less thymic cells. In addition, the RTOCs established with low numbers of TSCs, mainly those containing 0.085 × 10^6^ TSCs, exhibited significantly reduced proportions of DP (CD4^+^CD8^+^) cells and increased percentages of CD4^+^ thymocytes. In addition, there were higher frequencies of TCRαβ^hi^ thymocytes, including both TCRαβ^hi^CD4^+^ and TCRαβ^hi^CD8^+^ cells, but no differences in the percentages of total positively selected thymocytes (TCRαβ^hi^CD69^+^) were seen, although the frequencies of total thymic Treg cells increased (19).

## Conclusions and Further Research

In summary, these results support our proposal that reduced, but not total, disappearance of T-TEC crosstalk, due to alterations in the formed thymic epithelial network formed by low numbers of TECs may be enough for supporting T-cell differentiation; below those levels a normal T lymphopoiesis seems to be impossible. Nevertheless, functional studies and the determination of the condition of the negative selection in these RTOCs, not analyzed in these preliminary studies, are necessary to accomplish these results. However, it seems obvious that to establish the minimal numbers of thymocytes and TECs to obtain functional immuno-competent cells may represent a promising tool to modulate central tolerance and to avoid autoimmunity.

## Author Contributions

SM-H, JG-C, and AGZ: investigation and manuscript writing. AGZ: funding acquisition. All authors accept the published version of the manuscript.

## Conflict of Interest

The authors declare that the research was conducted in the absence of any commercial or financial relationships that could be construed as a potential conflict of interest.
